# Comparison of high tone therapy and transcutaneous electrical nerve stimulation therapy in chemotherapy-induced polyneuropathy

**DOI:** 10.1097/MD.0000000000020149

**Published:** 2020-05-08

**Authors:** Dagmar Schaffler-Schaden, Robert Sassmann, Tim Johansson, Simon P. Gampenrieder, Gabriel Rinnerthaler, Kathrin Lampl, Juergen Herfert, Christiane Lenzhofer, Yvonne T. Landkammer, Florian Rieder, Richard Greil, Maria Flamm, Josef Niebauer

**Affiliations:** a Institute of Physical Medicine and Rehabilitation; b Institute of General Practice, Family Medicine and Preventive Medicine; c Department of Internal Medicine III with Haematology, Medical Oncology, Haemostaseology, Infectiology and Rheumatology, Oncologic Center, Salzburg Cancer Research Institute - Laboratory for Immunological and Molecular Cancer Research (SCRI-LIMCR); d Institute of Sports Medicine, Prevention and Rehabilitation, Paracelsus Medical University Salzburg, Austria.

**Keywords:** cancer, CIPN 20, neuropathy, oncology, QLQ-C30, quality of life

## Abstract

**Introduction:**

Chemotherapy-induced peripheral neuropathy (CIPN) is a worldwide concern in patients receiving neurotoxic agents for cancer therapy. High tone external muscle stimulation is a promising therapeutic approach to alleviate symptoms of CIPN.

**Methods:**

This pilot study aims to investigate whether the application of home-based high-tone external muscle stimulation therapy (HTEMS) improves symptoms of CIPN. The trial is planned as a therapist- and assessor-blinded, 1:1 randomized controlled study. A total of 50 patients with chemotherapy-induced peripheral polyneuropathy will be included. All patients will perform therapy at home. Study participants will be allocated randomly to the HTEMS therapy (intervention group) or to the transcutaneous electrical nerve stimulation (TENS, control group), respectively, following a standardized therapy schedule. Compliance of participants can be verified by reading out the tool box. Outcomes will be evaluated at baseline and after 8 weeks of home-based therapy. The primary outcome includes improvement of CIPN according to the patient-reported EORTC QLQ-CIPN 20 questionnaire. Secondary outcomes are the patient-reported change in health-related quality of life and clinician-reported changes of vibration sensibility, tendon reflexes, temperature sensibility, perception of touch, and strength of the lower leg muscles. Further a safety- and process evaluation will be performed.

**Discussion:**

This pilot RCT aims to evaluate the impact of home-based HTEMS as compared to TENS in CIPN. There is a need for an effective treatment for CIPN and the results of this study are expected to possibly identify a novel and effective treatment strategy in the future.

## 1 Introduction

The use of chemotherapy has led to prolonged remission and survival in numerous cancer diseases. Chemotherapeutic agents have negative side effects and some of them are long-lasting. One of the most common adverse side effects of taxanes, platins, or vinca alkaloids is chemotherapy-induced peripheral neuropathy (CIPN). Prevalence of CIPN was reported in 30% to 40% of patients treated with neurotoxic chemotherapy and may be transient or permanent.^[1,2]^ CIPN appears predominantly as sensory neuropathy and affects the peripheral parts of the extremities in a “stocking and glove” distribution. It often presents with symptoms like paresthesia and numbness or tingling, but pain and motor symptoms can occur as well. Symptoms of CIPN may lead to dose reduction or even early cessation of chemotherapy and therefore may affect tumor control and survival in cancer patients. Furthermore, CIPN impairs quality of life (QoL), functional capacity, and increases annual costs of health care.^[3]^ Several factors have been identified which affect the risk of developing CIPN like smoking, low creatinine clearance, preexisting neuropathy or genetic predisposition^[4]^; however, etiology and underlying mechanisms are still controversial.

Currently, no evidence-based treatment (drug or non-drug therapy) is available for CIPN. Several approaches to alleviate peripheral neuropathy have been proposed, but evidence showing a benefit of these procedures regarding clinically relevant endpoints is scarce.^[5]^ Treatment with antidepressants, antiepileptics, topical ketamine, acupuncture, or magnetic field therapy failed to show significant relief so far.^[6,7]^ Although some promising results have been reported regarding the effects of nutraceuticals in the prevention or treatment of CIPN (magnesium, calcium, vitamins E and B6, glutamine, glutathione, N-acetyl cysteine, omega-3 fatty acids, and alpha lipoic acid), none of the agents has reproducibly shown effectiveness; moreover, acetyl-l-carnitine used in a RCT for taxane-induced neuropathy even markedly deteriorated CIPN in the study group.^[8,9]^ So far, only duloxetine was found to provide a moderate effect after 5 weeks.^[10]^ Many patients with chronic CIPN are referred to physical and exercise therapy. A recent systematic review analyzed the effects of specific exercise protocols on CIPN symptoms such as balance control, physical function, and QoL. However, the evidence is limited due to small sample sizes, heterogeneity among investigations (eg, type of exercise and durations), and use of outcome assessment methods.^[11]^ Hence, there is a strong need to implement more effective treatment regimes in CIPN.

In general, electrical sensory interventions are used for pain relief, muscle stimulation, and generating deep warmth in different tissues. However, a recent Cochrane review concludes that the evidence for transcutaneous electrical nerve stimulation (TENS) to relieve neuropathic pain in adults is poor.^[12]^ On the contrary, treatment with high-tone external muscle stimulation (HTEMS or high-tone therapy) seems a promising approach in the therapy of CIPN. The application of HTEMS has been shown to be more effective than TENS in the therapy of diabetic peripheral neuropathy.^[13,14]^ Furthermore, HTEMS demonstrated improvement in pain, discomfort, sleep disturbance, and QoL in patients with end-stage renal disease due to uremic peripheral neuropathy.^[15,16]^ The primary objective of this pilot RCT is to determine the efficacy of HTEMS therapy in CIPN compared to TENS therapy. Secondary objectives are the evaluation of process and safety of home-based HTEMS in patients with chemotherapy-induced peripheral neuropathy. The intervention group will receive HTEMS and the control group will receive TENS therapy. This pilot study aims to increase the feasibility of a successful RCT since a pilot trial can help to prevent a discontinued RCT in many cases.^[17]^

## 2 Objective

The primary objective is to evaluate the impact of home-based HTEMS in patients with chemotherapy-induced peripheral neuropathy compared to TENS. The primary outcome measure is the patient-reported EORTC QLQ-CIPN 20 questionnaire (Table 1).

**Table 1:**
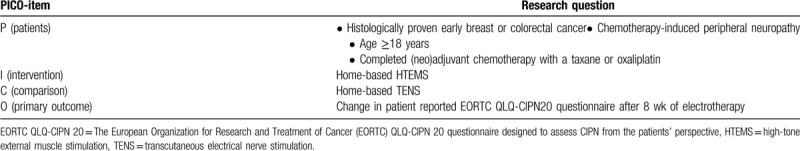
PICO research question of HTEMS trial.

Secondary objectives of this RCT are to evaluate the effectiveness of home-based high-tone therapy since a pilot study is useful to assure the feasibility of a future RCT:

1. Determination of acceptance of home-based electrotherapy (technical problems, user comfort)
2. Evaluation of recruitment, enrolment, randomization, completion, adherence, and attrition (withdrawal, dropout) rates
3. Evaluation of eligibility of recruited participants
4. Evaluation of data collection instruments
5. Collection of data on reasons for refusal to participate, reasons for nonadherence with the intervention and control therapy, and reasons for attrition

## 3 Methods/Design

**Figure 1:**
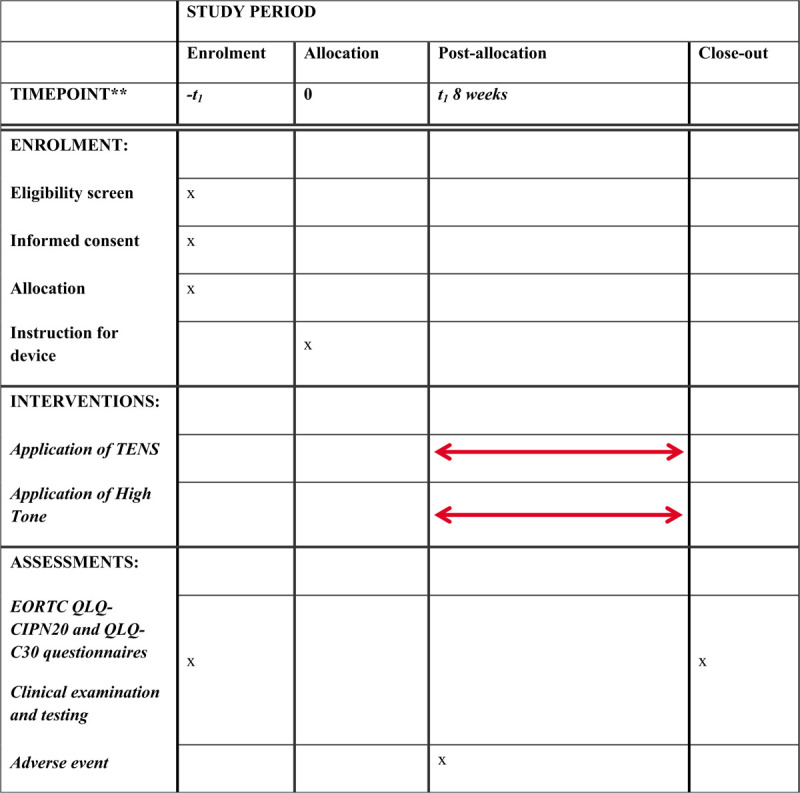
SPIRIT guideline diagram.

This is a single-blinded randomized controlled trial with an observation time of 8 weeks. The protocol has been written according to the Standard Protocol Items Recommendations for Interventional Trials (SPIRIT) Statement^[18]^ and will be reported according to the Consolidated Standards of Reporting Trials (CONSORT) Statement.^[19]^ The SPIRIT Checklist is provided in Additional file 1 and the SPIRIT Diagram is depicted in Figure 1.

### 3.1 Setting

The HTEMS-CIPN trial will be conducted at the University Hospital Salzburg and the Paracelsus Medical University in Salzburg, Austria, from January 2019 to December 2020.

### 3.2 Inclusion criteria

1. Age ≥18 years
2. Confirmed invasive cancer
3. Eastern Co-operative Oncology Group performance score 0 to 1
4. Completed neoadjuvant or adjuvant chemotherapy with a taxane or platin
5. Treatment with neurotoxic agent 4 to 24 weeks before enrolment
6. Clinical diagnosis of CIPN ≥Grade 1 according to Common Terminology Criteria for Adverse Events during or after completion of chemotherapy
7. Ability to complete questionnaires with or without assistance
8. Ability to understand the study protocol, its requirements, risks, and discomforts
9. Signed informed consent form

### 3.3 Exclusion criteria

1. Ongoing treatment with antitumor treatments with potential neurotoxic side effects (eg, platins, taxanes, vinca alkaloids, bortezomib, or thalidomide)
2. Completed chemotherapy with neurotoxic side effects other than taxane or platin
3. Preexisting clinically manifest peripheral neuropathy before initiation of chemotherapy (eg, caused by radiation or malignant plexopathy, lumbar or cervical radiculopathy, carpal tunnel syndrome, vitamin B12 deficiency, AIDS, monoclonal gammopathy, diabetes, heavy metal poisoning amyloidosis, syphilis, hyperthyroidism or hypothyroidism, inherited neuropathy, among others.)
4. Peripheral arterial occlusive disease >Grade 1
5. Skin conditions such as open sores preventing proper application of electrodes
6. Patients with implantable medical electronic devices (eg, pacemaker, implantable cardioverter defibrillator, catheter, and so on.)
7. Patients with reduced left ventricular ejection fraction (<55%) and/or history of myocardial infarction, cardiomyopathies, myocarditis or other myocardial diseases
8. Patients with cardiac arrhythmia
9. Patients with epilepsy
10. Patients with febrile illnesses or acute infectious diseases
11. Pregnancy

### 3.4 Patient identification and recruitment

The recruitment period covers 13 months (September 2019 to October 2020).

The Trial Steering Committee will supervise patient recruitment to ensure sufficient sample size in a stepwise patient recruitment approach.

1. Potential participants will be identified using a standardized admission form at the department of medical Oncology. The oncologists perform a precheck of the inclusion and exclusion criteria, inform potential participants about the study, and hand out the informed consent. The inclusion and exclusion criteria will be verified and informed consent will be obtained by the responsible physician at the Institute of Physical Medicine and Rehabilitation (PMR).
2. Baseline data collection and outcome assessment (clinical examination and patient questionnaire) will be performed at the Institute of PMR.
3. Randomization procedure and group allocation will be performed at the Institute of General Practice, Family Medicine, and Preventive Medicine.
4. Participants will be allocated to either the HTEMS or TENS group after completion of chemotherapy, respectively (consecutive sampling).
5. Instruction about the use of devices (intervention delivery) will be provided by a medical engineer at the Institute of PMR.
6. Participants are instructed to follow the treatment protocol as described in the section delivery of intervention.

Following strategies to fulfill the recruitment goal have been developed: presenting the study for staff members, trial fact sheets for staff members, close contact with oncologists responsible for the patient identification, regular reports on the progress of recruitment.

### 3.5 Randomization

The random allocation sequence will be generated using the random number generator available online. To ensure balance in the sample size across groups, we will perform stratified block randomization (ratio 1:1, block size 4). Subjects will be stratified by treatment delivery, taxane, and platin. To ensure concealment of allocation, randomization by computerized sequence generation will be performed as soon as patient recruitment is completed. After baseline data and outcome assessment, participants will consecutively be randomly assigned to the intervention or the control group. Thus, neither patients nor the responsible physician will know about the allocation to the HTEMS or TENS group at the time of recruitment.

### 3.6 Blinding

Physicians responsible for the clinical examinations and outcome assessment will be blinded. Due to the technical design of the intervention, participants and device instructors cannot be blinded. The analysts and outcome assessors will be kept blind to patient allocation.

### 3.7 Intervention

#### 3.7.1 Description and delivery of HTEMS

The primary aim of HTEMS is to activate the metabolism of the cells. HTEMS is based on the idea that not only active exercise such as sports improves the metabolism, but also “passive applications” in the form of external electrical muscle stimulation will have positive effects. In contrast to TENS therapy, HTEMS operates directly on the muscle in-depth, producing pleasant but intense and thus effective contractions. Additionally, HTEMS modulates amplitude and frequency simultaneously. The applied frequencies range continuously from 4.096 to 32.768 Hz. It is important to offer a broad spectrum of frequencies as different frequencies activate structures of different sizes. The electrical stimulation is applied by using conductive rubber electrodes. The electrodes are positioned proximally and distally on the lower limbs. During the treatment, the muscle is stimulated to contraction by intervals. One interval consists of 3-second ramp-up time (intensity rises), 3-second holding time (intensity maintained at maximum), and 3-second pause (no stimulation). HTEMS will be performed with a HiTOP 191 therapy device (gbo Medizintechnik, Rimbach, Germany), which is a 230 V power supply device. The first treatment is performed and supervised by a training therapist at the Institute of PMR. Frequency and amplitude (intensity) are adjusted according to the manufacturers’ instruction individually to a tolerable level causing muscle contraction so that neither pain nor discomfort is produced. The participant is educated for home-based therapy and the treatment should be used daily for 30 minutes at least 5 days per week for 8 weeks. No specific daily time points are given to avoid constraining patients or disturbing their individual rhythm. The intervention should be performed regularly at a time point adapted to the patient's daily routine. The therapy will start within 4 to 24 weeks after completed chemotherapy to reduce the effect of any known or unknown biases related to the treatment regime. Potential study participants will receive information about the study as soon as symptoms occur, with inclusion, randomization, and allocation occurring within 4 to 24 weeks after finishing chemotherapy. The intervention will last 8 weeks.

### 3.8 Controls

#### 3.8.1 Description and delivery of TENS

Participants who are allocated to the control intervention group will receive TENS therapy. TENS is mainly used for musculoskeletal and neuropathic pain treatment and also offers functional benefits. The device sends electrical impulses through conductive rubber electrodes to the skin of the impaired region. TENS is used in medical settings and can be self-administered by patients at home. In TENS only the frequency modulation is possible. The applied frequencies range from 2 to 120 Hz. The pain region must be in the middle of the 2 electrodes, with a maximum distance of 20 cm. Eligible participants will be identified in the same way as the HTEMS group. Randomization determines allocation. TENS will be performed with a DoloBravo therapy device 10-05 (MTR GmbH, Scheideggweg 7, 12277 Berlin, CE 0123), which is operated by a small 9 V block battery. A medical engineer will provide use instruction. The first treatment is performed and supervised by a training therapist at the Institute of PMR. Frequency (intensity) is applied according to the manufacturers‘ instructions. The participant is educated for home-based therapy and the treatment should be used daily for 30 minutes for at least 5 days per week during the 8 weeks of intervention. Additionally, participants‘ compliance will be checked by reading out the toolbox.

### 3.9 Baseline-examination

Shortly after consent is obtained, the study team will assess the following parameters and record all data in each patient's case report form (CRF):

1. age and sex
2. height and weight
3. diagnosis and co-morbidities
4. level of education
5. smoking behavior
6. chemotherapy schedule
7. CIPN/grading

Participants are asked to fill out the questionnaires (QLQ-CIPN 20 and QLQ 30) in paper form. Clinical examination will be performed by physicians at the Institute of PMR.

### 3.10 Follow-up, final examinations

Patients will be contacted by the study team after 1 week to identify any problems with the device. After 8 weeks of electrotherapy (HTEMS or TENS), the outcome assessments (physician-reported outcome and patient-reported outcome) from baseline will be repeated. Reporting of results will be in accordance with the principles of the Consolidated Standards of Reporting Trials (CONSORT) statement.^[19]^

### 3.11 Process evaluation and compliance

Participants‘ compliance in the intervention and the control group will be checked by reading out the toolbox of the HTEMS and TENS device. The minutes in use and intensity will be recorded for every device. Furthermore, patients’ diaries will be evaluated to check for accordance with the toolbox records.

### 3.12 Monitoring

A detailed monitoring and quality plan that complies with Good Clinical Practice guidelines will be developed. This monitoring and quality plan will involve standard operating procedures as well as documentation of any safety parameters related to electrotherapy. Additionally, this includes monitoring of the rate of recruitment, identifying and resolving data errors or omissions on the forms, monitoring for protocol violations, and looking for unanticipated events. Quality assurance includes checking of the case report forms for completeness and clarity, cross-checking with source documents, and clarification of administrative matters. Furthermore, the site monitors will assure that the study is conducted according to the protocol design and regulatory requirements. Complications and side effects will be documented. The number of patients who will not complete the full course of therapy and the reasons for this will be recorded. Since this is a pilot study and due to the sample size no interim analyses will be performed. The Institute of General Practice, Family Medicine, and Preventive Medicine is responsible for the monitoring and the monitor is independent from the sponsor and competing interests.

### 3.13 Potential harms and patient safety

According to current literature, it is not to be expected that complications or harm will be caused by electrotherapy. The observed side effects of HTEMS were nervousness and sleep disorders.^[13,15]^ Nevertheless, we will monitor potential harms. Undesirable or adverse effects of electrotherapy will be registered by the study team. The study team reports the events in the CRF as soon as they become aware of the event. They are responsible to judge whether the adverse event is associated with the intervention. Stopping criteria comprise: dysesthesia, aggravation of symptoms, adverse events with the devices, incompatibility of the electrodes, progress of underlying disease, or any new upcoming exclusion criteria during the intervention. Additionally, after inclusion study participants get a telephone number of a study team member to report any adverse events or other concerns. There are no concomitant care and interventions that are prohibited—usual care will continue during the study period. Insurance for the study participants is provided. Study data will be locked and only the study team will have access. Included patients are anonymized with a study identification number.

### 3.14 Primary outcome

#### 3.14.1 Patient-reported outcome

The EORTC QLQ-CIPN 20 contains 20 items assessing sensory (9 items), motor (8 items), and autonomic symptoms (3 items), using a 4-point Likert scale (1 = “not at all,” 2 = “a little,” 3 = “quite a bit,” and 4 = “very much”). All scale scores are linearly converted to a 0 to 100 scale (0 = no sensory impairment, 100 = worst sensory impairment).^[20]^

### 3.15 Secondary outcomes

#### 3.15.1 Patient-reported outcome

The EORTC QLQ-C30 questionnaire is an integrated system for assessing the QoL of cancer patients participating in clinical trials. All of the scales and single-item measures range in score from 0 to 100.^[21]^

#### 3.15.2 Clinician-reported outcome

Baseline assessment is performed within 4 to 24 weeks after finishing the chemotherapy. Follow-up assessment is performed 8 weeks after intervention.

We use a standardized routine clinical test battery in our clinical examination containing the following assessment methods:

1. Vibration sensibility: evaluated by the use of a graduated Rydel-Seiffer tuning fork (128 Hz) with a scale from 0 to 8. Due to age-related neural deconditioning, values ≤4 are considered pathological for patients ≥60 years’ old, for patients <60 years’ old, scoring ≤5 is regarded as pathological.^[22,23]^
2. Tendon reflex: Achilles tendon and patellar tendon reflex is assessed with a reflex hammer and graded on a 5-point scale of 0 to 4 with 0 being no response, 1+ being diminished/low normal, 2+ being average/normal, 3+ being brisker than average/possibly indicative of disease, and 4+ being very brisk, hyperactive, with clonus.^[24]^
3. Temperature sensibility: Assessment by using TipTherm (tip therm GmbH, Düsseldorf, Germany). The examiner places the 2 circular end faces of the instrument alternately and in an irregular sequence on the back of the patient's foot and asks for the sensory impression: cold or less cold? Only correct answers suggest an intact temperature discrimination capability. Incorrect answers or uncertainties are to be understood as temperature sense—disturbance at the investigation site.
4. Perception of touch: stroking patients’ upper legs, lower legs, and feet to detect reduced or altered sensation due to demyelination or axonal degeneration.^[22]^
5. Strength of the lower muscles is assessed by asking the patient to perform toe standing/walking and heel standing/walking on both feet (possible, not possible)

### 3.16 Outcome Assessment

For outcome assessors, the treatment allocation remains blinded. The QoL disease-specific CIPN20 questionnaire and EORTC QLQ-C30 questionnaire are provided in the patient's language.

### 3.17 Data management

Baseline and follow-up data will be recorded pseudonymized using a CRF. Forms will be checked all for completeness and plausibility. In case of missing or implausible data, the responsible outcome assessor will be contacted.

### 3.18 Sample size

Since this is a pilot trial, a formal sample size calculation was not performed. We aimed to recruit 50 patients as that was the sample size that we estimated would be feasible within this pilot project to provide sufficient scientific validation. Pilot studies are important to be confident that the intervention can be delivered as intended. It will also allow better assumptions about effect sizes and variability.

### 3.19 Statistical analysis

Statistical analyses are elaborated in a prespecified statistical analysis plan. The primary endpoint is a change in the EORTC-QLQ-CIPN 20 score during the 8 weeks of intervention. For our primary endpoint, we will use an intention-to-treat analysis according to the CONSORT statement.^[19]^ Data that are missing at completely random (unrelated to any variable in the data set) or at random (related to some variable, but not to the outcome itself) will be imputed.^[25]^ If missing items in questionnaires exist, the patient's previous responses to the same item will be used.^[21]^ Further analyses will include a per-protocol analysis (primary and secondary outcomes) to test the consistency of intervention effects across important subgroups, for example, type of chemotherapy. Furthermore, we will analyze the intensity of intervention (compliance) and the effect on the primary endpoint (eg, comparison of infrequently and frequently use of device). A minimum use on 5 of 7 days per week is required. All tests are 2-tailed, and a 5% probability level is considered as significant. Statistical analyses are carried out after the end of the trial, with no interim analysis planned. All statistical analyses are performed with IBM SPSS Statistics 23.0.

### 3.20 Trial management and roles of study team

A Trial Steering Committee, consisting of a doctor in physical medicine, a training therapist, an oncologist, a general practitioner, and an expert in statistics, will supervise the trial. Their tasks provide overall supervision and ensure that the trial is conducted consistently. The Trial Steering Committee will discuss the protocol regularly every 6 weeks and at the end of the trial. This committee is further responsible to ensure adequate enrolment of participants, discuss modification or cessation of the trial as appropriate and reviews any case of reported undesirable effects.

## 4 Discussion

This pilot study aims to evaluate the efficacy of home-based HTEMS in CIPN and to determine the feasibility of a further large-scale RCT. To date, little research on high-tone therapy in CIPN has been carried out, even if this approach is widely used in clinical practice. We suppose that a structured treatment regime with high-tone therapy can improve patient care and symptom management of CIPN. Based on clinical observations, the effect of HTEMS on symptoms of CIPN seems promising. Although CIPN affects many patients with cancer, evidence for an effective therapy is still lacking. We cannot completely rule out that the therapy response in this pilot study is different depending on the type of chemotherapy administered. Oxaliplatin and paclitaxel can have different effects regarding the temporal course and localization of symptoms. Nevertheless, we decided to include different groups of patients. One reason is that there is no mandatory relation between the effect of HTEMS and the administered substance. CIPN causes predominantly sensory symptoms; motor or autonomic symptoms are much less common. Typically, oxaliplatin induces an acute sensory neurotoxicity with rapid onset of cold-induced distal dysesthesia and/or paraesthesia. The symptoms occur mostly during or shortly after administration and are transient and mild. With prolonged oxaliplatin treatment, a cumulative sensory peripheral neuropathy develops causing superficial and deep hypaesthesia and functional impairment. Furthermore, oxaliplatin affects hands more severely than feet.^[2]^ Neuropathy caused by taxanes develops cumulatively over time and typically improves after chemotherapy cessation. Oxaliplatin-related symptoms, in contrast, may get worse for several months after stopping chemotherapy, a phenomenon called “coasting-effect.”^[2]^ In this pilot study, we will include only patients with persisting CIPN 4 to 24 weeks after finishing the neurotoxic chemotherapy to exclude patients with spontaneous remission of sensory neuropathy.

## 5 Conclusion

In daily clinical routine, good results have been achieved with the application of electrotherapy for CIPN. This pilot study can provide important evidence about the therapeutic effects of home-based HTEMS in CIPN, which is a worldwide concern in oncologic patients. Since HTEMS has hardly any side effects, it seems to be a promising approach in the therapy of CIPN.

## Acknowledgments

The authors thank Sanitaetshaus Lambert GmbH, Salzburg, for the free provision of the medical devices.

Protocol Version 1, 8 Sept 2019.

## Author contributions

**Conceptualization:** Robert Sassmann, Tim Johansson, Kathrin Lampl, Juergen Herfert, Richard Grell, Maria Flamm, Josef Niebauer.

**Data curation:** Robert Sassmann.

**Formal analysis:** Tim Johansson.

**Funding acquisition:** Robert Sassmann, Juergen Herfert, Dagmar Schaffler-Schaden, Yvonne Landkammer.

**Investigation:** Robert Sassmann, Kathrin Lampl, Christiane Lenzhofer, Yvonne Landkammer, Florian Rieder.

**Methodology:** Simon Gampenrieder.

**Project administration:** Dagmar Schaffler-Schaden, Tim Johansson.

**Resources:** Simon Gampenrieder, Juergen Herfert, Florian Rieder.

**Supervision:** Christiane Lenzhofer, Richard Greil, Maria Flamm, Josef Niebauer.

**Validation:** Tim Johansson, Simon Gampenrieder.

**Writing – original draft:** Dagmar Schaffler-Schaden, Tim Johansson.

**Writing – review & editing:** Dagmar Schaffler-Schaden, Robert Sassmann, Tim Johansson, Simon Gampenrieder, Kathrin Lampl, Juergen Herfert, Christiane Lenzhofer, Yvonne Landkammer, Florian Rieder, Richard Greil, Maria Flamm, Josef Niebauer.
